# The IL33 receptor ST2 contributes to mechanical hypersensitivity in mice with neuropathic pain

**DOI:** 10.1186/s13041-021-00752-3

**Published:** 2021-02-17

**Authors:** Junting Huang, Vinicius M. Gadotti, Zizhen Zhang, Gerald W. Zamponi

**Affiliations:** 1 Department of Anatomy and Neurobiology, Zhongshan School of Medicine, Sun Yat-Sen University, Guangzhou, 510080 Guangdong China; 2 Department of Physiology and Pharmacology, Cumming School of Medicine, Hotchkiss Brain Institute and Alberta Children’s Hospital Research Institute, University of Calgary, Calgary, AB Canada

**Keywords:** TKR2, NLRP3, Pain, Spared nerve injury, IL33

## Abstract

Pathogen infection triggers pain via activation of the innate immune system. Toll-like receptors (TLRs) and Nod-like receptors (NLRs) are the main components of innate immunity and have been implicated in pain signaling. We previously revealed that the TLR2-NLRP3-IL33 pathway mediates inflammatory pain responses during hyperactivity of innate immunity. However, their roles in neuropathic pain had remained unclear. Here we report that although knockout of TLR2 or NLRP3 does not affect spared nerve injury (SNI)-induced neuropathic pain, intrathecal inhibition of IL33/ST2 signaling with ST2 neutralizing antibodies reverses mechanical thresholds in SNI mice compared to PBS vehicle treated animals. This effect indicates a universal role of IL33 in both inflammatory and neuropathic pain states, and that targeting the IL33/ST2 axis could be a potential therapeutic approach for pain treatment.

Pathogen infection initiates host defense via activation of the pattern recognition receptors such as Toll-like receptors (TLRs) and nod-like receptors (NLRs) in the innate immune system [1–3]. This process typically induces a pain response as alarming signals to prevent further tissue damage or potential injury. Moreover, a number of different TLRs and several NLRs have been implicated in various chronic pain states [4–8], indicating an intimate interaction between innate immunity and pain. We have recently reported that activation of innate immunity through intraplantar injection of either Complete Freund’s Adjuvant (CFA), or the specific TLR2/6 heterodimer ligand FSL1 triggers pain responses by increasing interleukin33 (IL33) levels in both the paw and the dorsal root ganglia (DRG) [9]. In both cases, blocking the IL33 receptor ST2 via intrathecal delivery of functional neutralizing antibodies mediated analgesia. The responses to CFA and FSL1 were abolished in TLR2 receptor null mice, whereas NLRP3 null mice continued to exhibit mechanical hypersensitivity in responses to CFA, but not FSL1 [9]. Furthermore, we showed that IL33 was a key factor in priming inflammatory pain responses. Collectively, our findings suggested that that IL33 is a critical mediator of acute and chronic inflammatory pain states. Importantly, we did not observe sexual dimorphism with regard to this pathway. Previous literature has revealed that IL33 is involved in mouse models of neuropathic pain [10, 11]. We therefore asked whether TLR2, NLRP3 and IL33 signaling are important for the development of chronic pain resulting from peripheral nerve injury.

We first examined the importance of TLR2 and NLRP3 in the development of mechanical hyperalgesia resulting from spared nerve injury (SNI) of the sciatic nerve in male mice. Wild type mice, TLR2 null mice and NLRP3 null mice were subjected to SNI as previously described, and mechanical paw withdrawal thresholds were examined [12, 13]. Briefly, mice were individually placed in a plexiglass chamber over a wire mesh floor and were habituated in chambers for a minimum of one hour prior to testing, and mechanical paw withdrawal threshold was measured using a digital plantar aesthesiometer (DPA, UgoBasile, Varese, Italy). The DPA was placed under the hind paw to allow direct stimulation of the plantar surface with the filament. Withdrawal thresholds were examined 3–5 times with interceding intervals. Compared to sham operated animals, WT mice subjected to SNI showed a significant drop in mechanical withdrawal thresholds when assessed 14 days after nerve injury (Fig. 1a). In contrast, TLR2 null mice and NLRP3 null mice still displayed mechanical hypersensitivity that was statistically indistinguishable from that of WT animals (Fig. 1a). Hence, we conclude that neuropathic pain requires neither the activation of TLR2 receptors nor the NLRP3 inflammasome, in contrast to what is observed with inflammatory pain states [9]. We then tested whether IL33 signaling at the spinal/DRG level is involved in neuropathic pain states. For this purpose, we performed SNI or sham operations, and then 14 days later we measured a baseline of paw withdrawal thresholds, followed by repeated treatment of ST2 receptor neutralizing antibody or PBS control via intrathecal injection (300 ng/10 μl, mouse ST2/IL33R antibody, R&D, MAB10041, delivered three hours prior to testing) (Fig. 1b). As shown in Fig. 1b, the ST2 receptor neutralizing antibody resulted in a partial, but robust and statistically significant increase in paw withdrawal thresholds in male mice. The functional ST2 antibody had no effect in sham operated male mice (mechanical withdrawal threshold—Baseline: 10.3 ± 0.7 g; ST2-AB: 10.3 ± 0.4 g, n = 7). We also examined the effect of the ST2 receptor neutralizing antibody in female mice. As shown in Fig. 1c, SNI triggered mechanical hypersensitivity in female mice that was not affected by intrathecal delivery of PBS, but was attenuated upon intrathecal delivery of the ST2 receptor antibody. Hence, there are no sex differences in the action of IL33, which fits with our previous observation that TLR2 signaling (which is upstream of the IL33/ST2 pathway) is equally important in male and female mice [9].

**Fig. 1:**
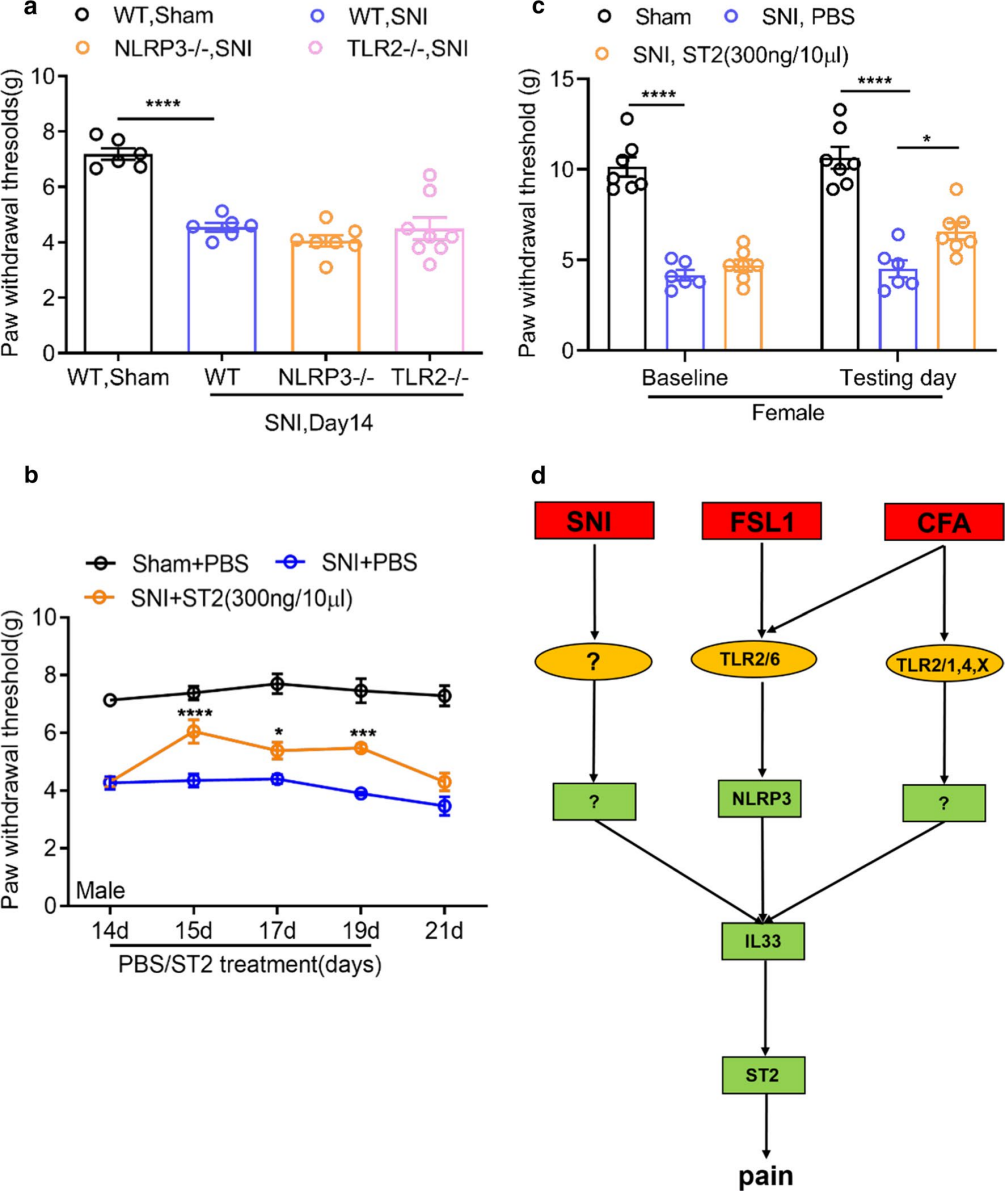
Role of TLR2, NLRP3 and IL33 in SNI induced mechanical hypersensitivity in mice. **a** Mechanical paw withdrawal thresholds in male WT, TLR2−/− and NLRP3−/− mice subjected to SNI (n = 6–8). Data are presented as mean ± S.E.M, ****P < 0.0001, One-way ANOVA with Bonferroni’s correction. **b** Effect of ST2 neutralizing antibody on paw withdrawal thresholds in nerve injured male mice (n = 6). Baseline was measured 14 days after injury, and PBS or ST2 antibody were delivered on days 15, 17, and 19. Data are presented as mean ± S.E.M, *P < 0.05, ***P < 0.001, ****P < 0.0001. Two-way ANOVA with Bonferroni’s post-hoc test (i.t: intrathecal). **c** Effect of intrathecal delivery of the ST2 receptor neutralizing antibody on mechanical withdrawal threshold in female SNI mice compared to a PBS control solution. Data were acquired on day 14 (baseline—no drugs) and 15 (testing day—PBS or ST2 antibody) after nerve injury. *P < 0.05 (ANOVA). **d** Schematic representation of pathways or IL33 induced pain signaling in response to FSL1, CFA and SNI

Altogether, our data fit with the notion that IL33 is upregulated in the DRG of nerve-injured mice, and suggest that IL33 interactions with its receptor are an important contributor to mechanical hypersensitivity after SNI. Hence, FSL1, CFA and SNI-mediated pain responses share a common endpoint with IL33. We did not examine possible effects of the ST2 receptor antibody at later time points (i.e., several weeks) after nerve injury, and we thus cannot rule out the possibility that other IL33 independent processes may be at play at such a stage.

Our previous work has shown that the ST2 receptor is expressed on sensory neurons, and that these neurons respond to IL33 with increased activity after peripheral priming with FSL1 [9]. Furthermore, IL33 levels were found to be elevated in both the hind paw and the DRG in FSL1 treated animals. Finally, direct delivery of IL33 at both spinal and peripheral sites resulted in pronounced thermal and mechanical hypersensitivity, and the duration of the spinally mediated response was greatly augmented by peripheral IL33 priming [9]. At this point, it is unclear if nerve injury-induced hypersensitivity involves a similar priming step. FSL1 is a selective TLR2/6 agonist, and consequently knockout of TLR2 abrogates the effects of FSL1 with regard to pain sensitization and IL33 production (see [9]). While the proalgesic effects of CFA and FSL1 were both absent in TLR2 null mice, NLRP3 null mice showed greatly attenuated pain responses in the FSL1 model but not the CFA model [9]. These data indicate that CFA may engage a parallel pathway for IL33 production that bypasses the NLRP3 inflammasome (see Fig. 1d). The effects of SNI on mechanical hypersensitivity on the other hand did not require the presence of TLR2 or NLRP3, and yet, spinal IL33 signaling appears to be important. This then suggests that cellular and molecular pathways that lead to IL33 production in inflammatory pain are distinct from that of neuropathic pain conditions (Fig. 1d), and further experimentation will be needed to decipher them. Possibilities include inflammatory mediators that are released after nerve injury, and macrophage infiltration into the DRG that is known to occur after peripheral nerve injury [14]. Nonetheless, the findings from our previous study combined with what is presented here suggest that interfering with IL33 signaling at the spinal level may serve as a new strategy against both inflammatory and neuropathic pain in a range of chronic pain conditions.

## Data Availability

All data generated or analysed during this study are included in this published article.

## Abbreviations

CFA: Complete Freund’s adjuvant
SNI: Spared nerve injury
IL33: Interleukin33
TLR: Toll-like receptor
NLR: Nod-like receptor
PBS: Phosphate buffered saline
